# Durable reprogramming of neutralizing antibody responses following Omicron breakthrough infection

**DOI:** 10.1126/sciadv.adg5301

**Published:** 2023-07-21

**Authors:** Wen Shi Lee, Hyon-Xhi Tan, Arnold Reynaldi, Robyn Esterbauer, Marios Koutsakos, Julie Nguyen, Thakshila Amarasena, Helen E. Kent, Anupriya Aggarwal, Stuart G. Turville, George Taiaroa, Paul Kinsella, Kwee Chin Liew, Thomas Tran, Deborah A. Williamson, Deborah Cromer, Miles P. Davenport, Stephen J. Kent, Jennifer A. Juno, David S. Khoury, Adam K. Wheatley

**Affiliations:** ^1^ Department of Microbiology and Immunology, University of Melbourne, Peter Doherty Institute for Infection and Immunity, Melbourne, VIC, Australia.; ^2^ Kirby Institute, University of New South Wales, Kensington, NSW, Australia.; ^3^ Victorian Infectious Diseases Reference Laboratory, The Royal Melbourne Hospital at The Peter Doherty Institute for Infection and Immunity, Melbourne, VIC, Australia.; ^4^ Department of Infectious Diseases, The University of Melbourne at the Peter Doherty Institute for Infection and Immunity, Melbourne, VIC 3000, Australia.; ^5^ Melbourne Sexual Health Centre and Department of Infectious Diseases, Alfred Hospital and Central Clinical School, Monash University, Melbourne, VIC, Australia.

## Abstract

Severe acute respiratory syndrome coronavirus 2 (SARS-CoV-2) breakthrough infection of vaccinated individuals is increasingly common with the circulation of highly immune evasive and transmissible Omicron variants. Here, we report the dynamics and durability of recalled spike-specific humoral immunity following Omicron BA.1 or BA.2 breakthrough infection, with longitudinal sampling up to 8 months after infection. Both BA.1 and BA.2 infections robustly boosted neutralization activity against the infecting strain while expanding breadth against BA.4, although neutralization activity was substantially reduced for the more recent XBB and BQ.1.1 strains. Cross-reactive memory B cells against both ancestral and Omicron spike were predominantly expanded by infection, with limited recruitment of de novo Omicron-specific B cells or antibodies. Modeling of neutralization titers predicts that protection from symptomatic reinfection against antigenically similar strains will be durable but is undermined by new emerging strains with further neutralization escape.

## Teaser

Omicron breakthrough infection elicits durable neutralising activity by recalling cross-reactive vaccine-elicited memory B cells.

## INTRODUCTION

Severe acute respiratory syndrome coronavirus 2 (SARS-CoV-2) continues to cause substantial morbidity and mortality. While now licensed vaccines based on the ancestral strain of SARS-CoV-2 (Wuhan-Hu-1) are effective at preventing severe coronavirus disease 2019 (COVID-19), they have reduced effectiveness at preventing infection with novel variants that escape vaccine-elicited neutralizing antibodies. The Omicron variant is highly antigenically distinct and rapidly outcompeted the Delta variant to become the dominant global strain in early 2022. Various sublineages of Omicron (BA.1, BA.2, BA.4, BA.5, XBB, BQ.1.1, and others), each with different degrees of antibody evasion, have since emerged in successive waves (*1*, *2*). The high transmissibility and immune evasion of Omicron, in tandem with waning of vaccine-elicited immunity, have resulted in increasing frequencies of “breakthrough” infections of vaccinated individuals. At a population level, immunity against SARS-CoV-2 is thus becoming increasingly complex, with variable dosing and types of vaccines, infection with distinct variants, or a combination of both (hybrid immunity).

Recent studies of antibody and memory B cell responses following breakthrough infection with Delta or Omicron BA.1 have established rapid anamnestic recall of spike-specific antibody responses, reactivation of spike-specific memory B cells, and differentiation of antibody-secreting cells (*3*–*5*). Breakthrough Omicron infection in individuals with two prior vaccine doses has also been associated with increases in the breadth of serum neutralizing antibody activity compared to those receiving a third dose of an ancestral vaccine (*3*, *6*). Increased neutralizing breadth could be derived from de novo antibody responses against neo-epitopes within Omicron spike or, alternatively, the selective reexpansion of cross-reactive memory B cells established during vaccination. Here, we intensively examined the early kinetics of recalled immunity following Omicron BA.1 or BA.2 breakthrough infections, as well as profiled the durable changes in serological neutralization breadth following recovery. We find that breakthrough infections, despite mild disease course, were highly efficient at both recalling spike-specific memory responses established by prior immunization, as well as generating novel responses to the viral nucleocapsid (N). While BA.1 or BA.2 neutralizing titers were low or undetectable at early time points following infection, these responses expanded robustly and demonstrated breadth against the BA.4 variant, although neutralization against the more escaped XBB and BQ.1.1 variants was limited. Longitudinal follow-up revealed a stable maintenance of Omicron-specific neutralizing activity, which we then modeled to estimate the protective window against reinfection with the same or novel variants with further immune escape. Understanding the impact of periodic infection with increasingly distinct SARS-CoV-2 variants upon the durability and breadth of antibody and memory B cell immunity will be critical to informing optimal design and deployment of COVID-19 vaccines to maximize the population-level protection against future variants.

## RESULTS

### BA.1 or BA.2 breakthrough infection drives high viral loads and is immunogenic despite mild disease course

**Fig. 1.**
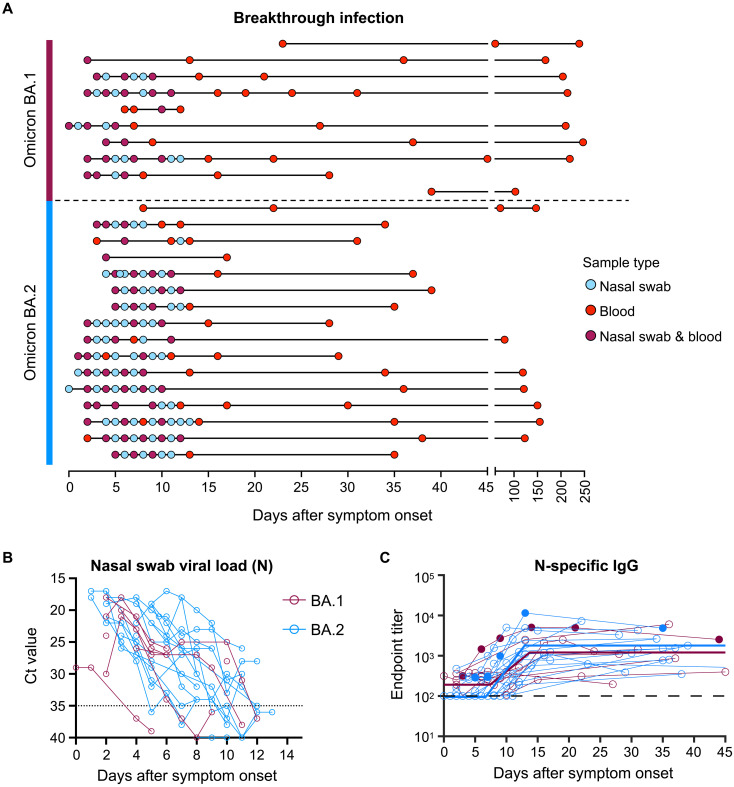
Viral load kinetics and seroconversion to N following Omicron BA.1 and BA.2 breakthrough infection. (**A**) Schematic of longitudinal sample collection following breakthrough infection of vaccinated individuals with Omicron BA.1 (*n* = 10) or BA.2 (*n* = 16). Each line represents a single subject, and each point represents a sample collection (blue, nasal swab; red, blood; purple, both nasal swab and blood). (**B**) Kinetics of SARS-CoV-2 viral load in nasal swabs measured by quantitative polymerase chain reaction (qPCR) of the nucleocapsid (N) gene. (**C**) Kinetics of plasma IgG titers against SARS-CoV-2 N following breakthrough infection with BA.1 (red) or BA.2 (blue). Subjects with previous SARS-CoV-2 infection are depicted in closed circles. The thick lines represent the mean estimate from the piecewise linear regression model using the estimated parameters.

Twenty-six vaccinated individuals (3 with two prior vaccines, 21 with three prior vaccines, and 2 previously infected with ancestral virus and subsequently vaccinated) were recruited following Omicron breakthrough infection that occurred at a median of 95 days [interquartile range (IQR), 78 to 124] after receiving their last COVID vaccine dose (table S1). All individuals reported a mild but symptomatic disease course. Omicron BA.1 infection was confirmed using whole genome sequencing for 8 individuals, and BA.2 was confirmed for 15 individuals, with the remaining presumptively designated BA.1 (2 individuals) or BA.2 (1 individual) infections based on the prevalent variants circulating at the time. Serial blood samples and nasal swabs were obtained up to 247 days of follow-up, with intensive sampling during the acute infection phase (Fig. 1A).

SARS-CoV-2 viral load in nasal swabs was measured via quantitative polymerase chain reaction (qPCR) of the N gene (Fig. 1B). Omicron BA.1 and BA.2 breakthrough infection showed similar viral kinetics, with most individuals displaying peak viral load upon recruitment at around 1 to 3 days after symptom onset. Median peak cycle threshold (Ct) values were consistent with previous reports demonstrating robust viral replication during Omicron breakthrough infection despite prior immunization (*7*, *8*). Nineteen of the 21 individuals in our cohort had no prior documented COVID-19 infection and were immunized with COVID-19 vaccines encoding only the spike antigen (BNT162b2, ChAdOx1 nCoV-19, mRNA-1273, and NVX-CoV2373) (table S1). Supporting this, serological responses against SARS-CoV-2 N were at low or undetectable levels at the earliest time points sampled (Fig. 1C). However, a clear and consistent expansion of N-specific IgG was observed in plasma samples from ~7 days after symptom onset, with similar trajectories for both BA.1 and BA.2 breakthrough infections (table S2). Overall, individuals with Omicron breakthrough infection in our cohort exhibited marked viral replication in the upper respiratory tract and seroconverted to N.

### Durable boosting of neutralizing antibody responses following BA.1 or BA.2 breakthrough infection

**Fig. 2.**
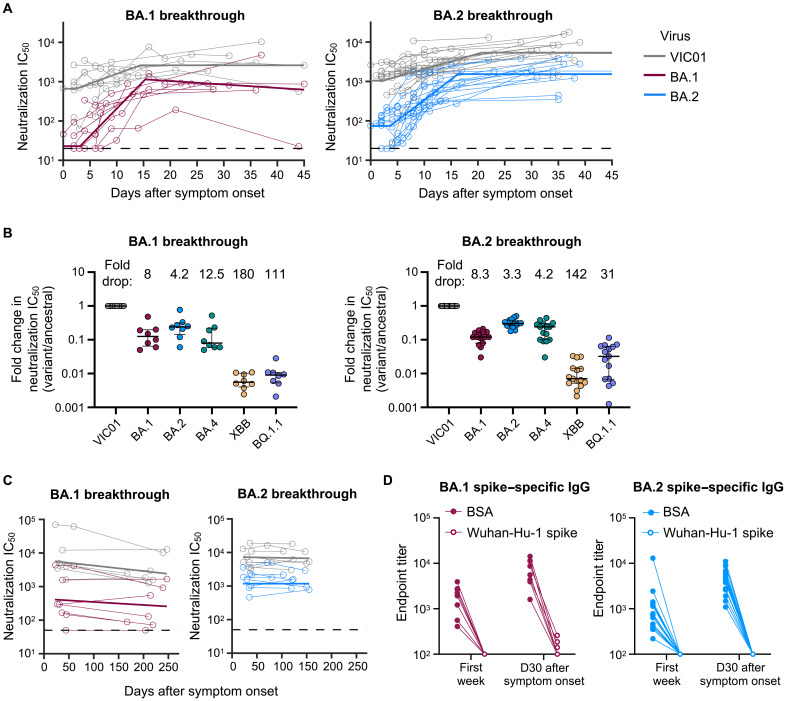
Omicron BA.1 and BA.2 breakthrough infection rapidly recalls neutralizing antibodies that are broad and durable. (**A**) Kinetics of plasma neutralization activity following breakthrough infection against ancestral VIC01 or matched infecting Omicron BA.1 and BA.2 strains. Thick lines represent the mean estimate from the piecewise linear regression model using the estimated parameters. Plasma neutralization activity was measured using a live virus neutralization assay against SARS-CoV-2 clinical isolates in human embryonic kidney–293T cells transduced with ACE2 and TMPRSS2. (**B**) Neutralization mediated by BA.1 and BA.2 breakthrough plasma against ancestral VIC01, Omicron BA.1, BA.2, BA.4, XBB, and BQ.1.1 strains at a median of 34 days after symptom onset. Data are presented as fold change in neutralization IC_50_ (variant/ancestral), with lines and error bars depicting median ± IQR, and fold drop in neutralization IC_50_ from ancestral virus shown above each variant. (**C**) Longitudinal decay kinetics of plasma neutralization activity following breakthrough infection against ancestral VIC01 or matched infecting Omicron BA.1 or BA.2 strains up to 4 to 7 months after symptom onset. The best fit decay slopes (thick lines) are depicted. (**D**) IgG antibody endpoint titers against BA.1 spike for BA.1 breakthrough subjects (red) and against BA.2 spike for BA.2 breakthrough subjects (blue) following preincubation with BSA control (closed circles) or ancestral Wuhan-hu-1 spike (open circles). D30, day 30.

At the earliest sampled time point following infection (<5 days after symptom onset), most subjects had robust serological neutralization activity against ancestral SARS-CoV-2 virus (VIC01) but low or undetectable neutralization titers against BA.1 or BA.2 (Fig. 2A). Neutralizing titers against the ancestral strain were rapidly recalled by infection, starting from days 3 to 4 onward and concomitant with rises in neutralization activity against the matched infecting strain. One BA.1-infected individual (CP69) had a transient rise in BA.1 neutralization activity from days 9 to 21, before waning to undetectable levels at day 44 after symptom onset. However, in all other subjects, recovery from infection was associated with robust boosting of BA.1 or BA.2 plasma neutralization activity.

We applied a piecewise linear regression model to parameterize the kinetics of antibody recall, including estimates of the initial period of delay, the rate of increase, and time to maximal titers (table S3). The initial delay phase before detectable increases in neutralizing activity against the homologous infecting strain was similar between BA.1- or BA.2-infected subjects, at 3.1 and 3.6 days, respectively, with a doubling time of 2.1 and 2.8 days thereafter. Robust expansion of BA.1 and BA.2 neutralizing activity against the infecting homologous strains was observed in both BA.1- and BA.2-infected cohorts (31- and 34.7-fold, respectively), contrasting with the more modest rise of neutralizing activity against VIC01 (5.4- and 15.6-fold, respectively).

We next examined the breadth of neutralizing antibody responses following breakthrough infection with a panel of Omicron variants including the more immune evasive BA.4, XBB, and BQ.1.1 variants. At around 1 month after symptom onset (median, 34 days; IQR, 28.5 to 36.5), individuals with BA.1 breakthrough infection had median neutralization titers of 1188 against VIC01, 190 against BA.1, 316 against BA.2, and 176 against BA.4, while individuals with BA.2 breakthrough infection had median titers of 5038 against VIC01, 616 against BA.1, 1468 against BA.2, and 936 against BA.4 (fig. S1). Similar to previous studies using live virus neutralization assays (*9*, *10*), plasma from both BA.1 and BA.2 breakthrough–infected subjects neutralized Omicron BA.2 better than Omicron BA.1. However, neutralization against the more recent and highly escaped XBB and BQ.1.1 variants was limited, with only two of the eight BA.1-infected individuals having neutralization activity above the limit of detection (LOD) for both viruses. BA.2-infected individuals had median titers of 76.5 against XBB (9 of 15 above LOD) and 383 against BQ.1.1 (12 of 15 above LOD). Neutralizing titers were consistently higher in subjects with BA.2 breakthrough infection; however, this is likely reflective of higher baseline titers in these individuals rather than any differential immunogenicity between BA.1 and BA.2. When normalized to ancestral VIC01 neutralization titers (Fig. 2B), the degree of escape relative to ancestral virus was consistent between the two cohorts for BA.1and BA.2 (~8- and ~3- to 4-fold drop, respectively). BA.2-infected individuals had lower fold drops in neutralization against BA.4, XBB, and BQ.1.1 (4.2-, 142-, and 31-fold, respectively), compared to that of BA.1-infected individuals (12.5-, 180-, and 111-fold, respectively).

The longevity of neutralizing antibody responses following breakthrough infection was examined in a subset of BA.1-infected (*n* = 7, days 167 to 247) and BA.2-infected subjects (*n* = 8, days 80 to 155) (table S1). After BA.1 infection, BA.1 neutralizing titers were stably maintained and readily detectable 5 to 8 months after infection, with a half-life of 334 days (Fig. 2C and table S4). An exception was a single subject (CP69) who did not have detectable titers after day 44. Neutralization against ancestral VIC01 decayed at a half-life of 183 days and remained high (>1:1000) for all individuals at the late time point. Durable BA.2-specific neutralizing responses were similarly observed in BA.2-infected subjects, with a half-life of 1050 days. Notably, four of the eight BA.2-infected individuals exhibited an increase in neutralizing activity between early and late time points, indicating that peak titers may have occurred more than 1 month after symptom onset (Fig. 2C and table S4). Neutralization against ancestral VIC01 virus was comparably durable and maintained over the 3- to 4-month period, with a half-life of 1216 days.

### Omicron BA.1 and BA.2 breakthrough infection recalls antibodies that are predominantly cross-reactive against ancestral SARS-CoV-2 spike

Robust and broad neutralizing responses against Omicron strains following breakthrough infection could derive from recalled immunological memory or comprise de novo BA.1- or BA.2-specific antibodies. We preincubated plasma samples with Wuhan-Hu-1 spike or a bovine serum albumin (BSA) control to sequester antibodies that bind to ancestral spike and probed for residual binding activity against BA.1 or BA.2 spike by immunoglobulin G (IgG) enzyme-linked immunosorbent assay (ELISA). In plasma samples taken during the first week after symptom onset, preincubation with ancestral spike eliminated any residual biding to the BA.1 and BA.2 spike, with no evidence for omicron-specific reactivity (Fig. 2D). However, after recovery (~1 month after symptom onset), low titers of antibodies specific for BA.1 alone were detected in three of the eight individuals. In contrast, no BA.2-infected subjects had antibodies specific for BA.2 alone. This demonstrates that antibody responses to BA.1 and BA.2 breakthrough infection are dominated by cross-reactive specificities that also bind to ancestral spike.

### Omicron BA.1 and BA.2 breakthrough infection primarily recalls memory B cells cross-reactive with ancestral SARS-CoV-2 spike

**Fig. 3.**
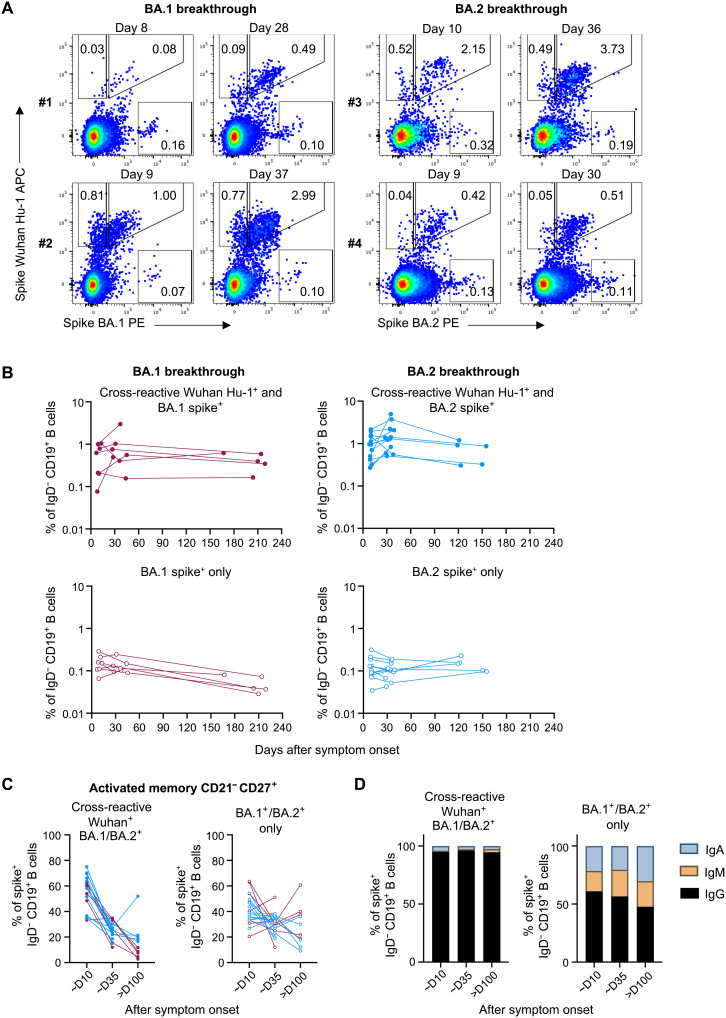
Omicron BA.1 and BA.2 breakthrough infection primarily recalls memory B cells that are cross-reactive against ancestral spike. (**A**) Representative flow cytometry plots of memory B cells (CD19^+^ IgD^−^) stained with ancestral (Wuhan Hu-1) and BA.1 or BA.2 fluorescent spike probes, from two individuals with BA.1 breakthrough infection and two with BA.2 breakthrough infection at around days 10 and 30 after symptom onset. APC, allophycocyanin. (**B**) Frequencies of memory B cells (CD19^+^ IgD^−^) from BA.1 breakthrough subjects (red) and BA.2 breakthrough subjects (blue) that are cross-reactive with Wuhan Hu-1 and BA.1/BA.2 spike (top) or memory B cells that only recognize BA.1/BA.2 spike (bottom) over time. (**C**) Frequencies of activated memory B cells (CD21^−^ CD27^+^) that are cross-reactive or specific for BA.1/BA.2 spike from BA.1 breakthrough subjects (red) and BA.2 breakthrough subjects (blue). (**D**) Antibody isotype distribution of cross-reactive or BA.1/BA.2 spike monospecific memory B cells.

In individuals with prior SARS-CoV-2 immunity established by ancestral spike vaccines, Omicron breakthrough infection could potentially broaden the memory B cell repertoire by eliciting B cells against neo-epitopes within Omicron spike. We therefore examined the recall of cross-reactive spike-specific memory B cells established primarily through prior vaccination or the elicitation of de novo Omicron-specific memory B cells. Spike-specific memory B cells within peripheral blood mononuclear cell (PBMC) samples were stained using a combination of fluorescent spike probes (ancestral Wuhan Hu-1 spike in combination with either BA.1 or BA.2 spike; gating in fig. S2). We observed substantial expansion of class-switched memory B cells (CD19^+^ IgD^−^) that were cross-reactive to SARS-CoV-2 ancestral spike and BA.1/BA.2 spike (Fig. 3A). Frequencies of cross-reactive memory B cells peaked around 1 month after symptom onset, before subsequently waning over 100 days of follow-up (Fig. 3B). We did not observe a notable expansion of monospecific memory B cells against either BA.1 or BA.2 spike (Fig. 3A). BA.1 and BA.2 monospecific memory B cells were on average 7- and 16-fold lower than their cross-reactive counterparts around 1 month after symptom onset, with frequencies remaining unchanged throughout the longitudinal sampling period (Fig. 3B).

A large proportion of cross-reactive memory B cells exhibited an activated phenotype (CD21^−^ CD27^+^) in PBMC samples taken ~day 10 after symptom onset (median, 58%), indicating efficient antigen recall during Omicron breakthrough infection, which later waned over the course of recovery (median, 14% at ~day 160) and transitioned to a resting memory phenotype (Fig. 3C and fig. S3). We observed a distinct lack of activation of BA.1 or BA.2 monospecific memory B cells, suggesting that these cells likely represent background binding or B cells with low affinity for spike (Fig. 3C). Similarly, we did not observe any expansion or contraction of BA.1 or BA.2 monospecific B cells in the nonmemory CD27^−^ subsets (fig. S3). Cross-reactive memory B cells were predominantly IgG^+^ at all time points, with minor subpopulations of IgA^+^ or IgM^+^ isotypes (Fig. 3D). In contrast, 45 to 60% of BA.1/BA.2 monospecific memory B cells were IgG^+^, which remained relatively unchanged after breakthrough infection, suggesting limited exposure to antigen in vivo. Overall, our data suggest that the cross-reactive memory B cells predominate the recall response, with Omicron-specific populations remaining unexpanded, following Omicron breakthrough infection.

### Estimated protection against reinfection following breakthrough infection is robust but moderated by immune escape

**Fig. 4.**
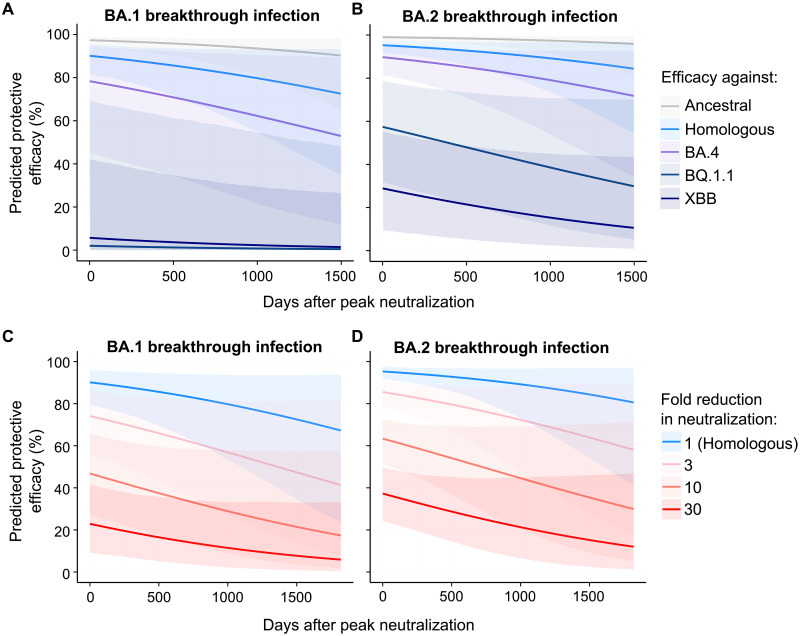
Modeling the protective efficacy against symptomatic SARS-CoV-2 reinfection following BA.1 and BA.2 breakthrough infection. (**A** and **B**) The predicted decay in efficacy against symptomatic reinfection after peak neutralization (about day 30 after symptom onset) based on the peak neutralization titers observed against ancestral virus, the homologous infecting Omicron variants and Omicron strains that individuals had not yet encountered (BA.4, XBB, and BQ.1.1) using the aggregate decay rate estimated. (**C** and **D**) The predicted decay in efficacy after peak neutralization titers given a theoretical loss of neutralization (i.e., 1-, 3-, 10-, or 30-fold loss of neutralization) to a new variant compared to the homologous neutralization titers after breakthrough infection [i.e., blue lines in (A) and (B)]. Lines are predicted efficacies and shaded regions indicate 95% confidence intervals of predicted efficacies.

The immunity gained at a population level via breakthrough infection remains unclear. Using the previously developed model for predicting protection from symptomatic infection based on neutralization titers (*11*), we estimated the durability of protection following Omicron breakthrough infection (Fig. 4, A and B). Because decay rates of neutralizing antibodies against the infecting strains were not significantly different across BA.1 or BA.2 breakthrough infection or across different variants (ancestral versus Omicron) (table S4), we pooled neutralization data for all subjects across all variants and fitted a linear mixed effects model to estimate an average decay rate of 880 days. Using the geometric mean peak neutralization titers from BA.1 and BA.2 breakthrough infection and the global decay rate, we predict that 70% protective efficacy against the homologous infecting strain would last approximately 4.5 years for BA.1 breakthrough and 7 years for BA.2 breakthrough. Similarly durable protective efficacy was estimated for the ancestral variant, lasting more than 8.5 and 10 years above 70% for BA.1 and BA.2 breakthrough, respectively. Forecasting protection to a variant to which participants had not been exposed, protective efficacy against BA.4 was predicted to be maintained above 70% for 705 and 1607 days for BA.1 and BA.2 breakthrough, respectively. Because of the marked fold drop in neutralizing titers, the projected protective efficacy against XBB and BQ.1.1 is substantially reduced, with BA.2-infected individuals predicted to have 28.9 and 57.4% protective efficacy against XBB and BQ.1.1 upon recovery, while BA.1-infected individuals only having 5.7 and 2% protective efficacy against XBB and BQ.1.1 upon recovery.

Given the continued emergence of Omicron sublineages with additional immune escape mutations, we next modeled the impact of immune evasion on the stability of protection against symptomatic reinfection. Notably, even at time points where plasma neutralization activity is maximal (~1 month after symptom onset), a novel variant with 3- or 10-fold reduction in neutralization would reduce the protective efficacy from 90.1 to 74.1 or 46.8%, respectively, for BA.1 hybrid immune individuals and from 95.3 to 85.5 or 63.4%, respectively, for BA.2 (Fig. 4, C and D). Overall, our modeling suggests that recovery from an Omicron breakthrough infection confers durable protection against closely related viral strains, but this protection can be rapidly subverted by the emergence of novel variants with increasing escape from neutralizing antibodies.

## DISCUSSION

The marked immune evasiveness and transmissibility of Omicron strains are driving increasing numbers of vaccine breakthrough infections worldwide, although the durability of immunity elicited by such infections remains unclear. Using intensive longitudinal sampling, we find that both BA.1 and BA.2 breakthrough infections drive expansion of neutralizing responses against the infecting strains from low or undetectable levels to high titers that were stably maintained from around 1 month after symptom onset. We show that only cross-reactive memory B cells were expanded by breakthrough infection, and the resulting antibody response was dominated by antibodies cross-reactive with ancestral spike, indicating that limited de novo responses were generated against neo-epitopes within BA.1 or BA.2 spike.

In line with recent studies (*3*, *12*), our results are suggestive of immune imprinting, with no evident increase in BA.1 or BA.2 monospecific B cells even up to 4 to 7 months after infection. Kaku *et al.* (*12*) reported that BA.1 breakthrough infection drove affinity maturation of cross-reactive B cells and antibodies toward Omicron BA.1 over time, although our data suggest that, mechanistically, this occurs through selective reexpansion of B cell memory. Supporting this, studies of the clonal repertoire of expanded cross-reactive memory responses after breakthrough infection show a strong conservation of vaccine-established clones and clonal families (*12*, *13*). In addition, there was little evidence of additional gains in Vgene somatic mutation in responding memory B cells (*13*), again suggesting that affinity maturation of the polyclonal antibody response toward Omicron spike is via selective expansion of higher affinity B cells instead of secondary germinal center activity. While the isolation of receptor binding domain (RBD)–specific monoclonal antibodies (mAbs) specific for the BA.1 RBD that do not cross-react with ancestral RBD has been reported, these comprised only a small fraction (median, 4%) of the response to RBD (*12*), confirming that neo-epitopes are poorly recognized during breakthrough infection. Immune imprinting is not constrained to breakthrough infections, as monovalent Omicron BA.1 or bivalent Beta/Delta mRNA vaccines also predominantly boost preexisting cross-reactive responses (*14*).

While superficially attractive, “overcoming” immune imprinting to generate responses against neo-epitopes may not actually be favorable for protection. Primary infection with Omicron elicits very limited neutralizing breadth against historical non-Omicron variants (*15*). Similarly, Omicron-specific mAbs isolated from BA.1, BA.2, or BA.5 breakthrough individuals that do not bind ancestral RBD exhibit very narrow breadth of recognition, with little to no neutralization activity against more recent Omicron variants including XBB and BQ.1.1 (*16*).

It remains unclear whether there is something unique about “hybrid immunity” (vaccination then infection) in terms of durable reprogramming of the antibody response. Epidemiological studies have indicated that hybrid immunity confers stronger and more durable protection against infection compared to vaccine- or infection-elicited immunity alone (*17*–*20*). In line with other reports (*6*, *21*), our data suggest that BA.1 and BA.2 breakthrough infection confers neutralization breadth that extend to the more immune evasive BA.4 variant, although neutralization against XBB and BQ.1.1 is limited. Studies of a third dose of an ancestral spike vaccine have similarly demonstrated expansion of Omicron-specific B cells and enhancement of neutralization breadth (*22*, *23*), although not to the same extent as breakthrough infection with BA.1 (*6*). It remains to be seen whether the bivalent ancestral and BA.1 or ancestral and BA.5 mRNA vaccines can elicit equivalent levels of neutralization breadth to Omicron breakthrough infection. Intriguingly, Muik *et al.* (*10*) showed that neutralization of BA.2 and BA.4/5 mediated by BA.2 breakthrough sera is highly dependent on antibodies against the N-terminal domain (NTD) of spike, while BA.1 breakthrough sera-mediated neutralization is almost exclusively dependent on RBD antibodies. The different antibody targets elicited by BA.1 versus BA.2 breakthrough infection may have implications for immunity toward future Omicron variants, depending on which region of spike (RBD and/or NTD) gains further escape mutations (*16*, *24*).

Both waning immunity and immune escape of new variants contribute to susceptibility of SARS-CoV-2 reinfection, although the relative contribution of these two factors is unknown. Here, we modeled the level of immune boosting and rate of antibody waning after breakthrough infection with BA.1 and BA.2 variants. Our analysis suggests robust and prolonged immunity to the homologous strain (70% protective efficacy for 4.5 and 7 years following BA.1 or BA.2 breakthrough infection). However, this protection is rapidly undermined by the emergence of more escaped variants. For example, a variant with a threefold reduction in neutralizing antibody titer would reduce protective efficacy by the same amount expected after 3.8 years of waning immunity. A novel variant with a 10-fold reduction in neutralizing antibody titer would reduce protective efficacy to 46.8 and 63.4% immediately following BA.1 or BA.2 breakthrough infection. This suggests that waning immunity likely plays a relatively small role in the ongoing susceptibility of SARS-CoV-2, especially with the emergence of XBB (BA.2-derived) and BQ.1.1 (BA.5-derived) variants that have displayed substantial fold drops in neutralization relative to ancestral virus in BA.1/BA.2 breakthrough cohorts (*16*, *25*, *26*). Our results suggest that protection against XBB and BQ.1.1 is limited following BA.2 infection (28.9 and 57.4% protective efficacy, respectively) and negligible following BA.1 infection. The higher neutralization activity and protection provided by BA.2 breakthrough infection against XBB and BQ.1.1 compared to BA.1 breakthrough could be due to XBB and BQ.1.1 being antigenically more similar to BA.2 than to BA.1 (*27*).

Repeated, sequential waves of Omicron outbreaks have driven substantial breakthrough infections and conferred “hybrid” immunity to much of the global population. Our data suggest that continuing spread of SARS-CoV-2 in this immune landscape will be less a function of waning immunity and, in large part, depend on the viral acquisition of further neutralization escape mutations.

## MATERIALS AND METHODS

### Human subjects and ethics

A cohort of previously vaccinated participants with breakthrough COVID-19 (either PCR or rapid antigen test positive) were recruited through contacts with the investigators and invited to provide serial blood and nasal swab samples following symptom onset (table S1), some of whom were previously described (*5*). Infecting variant was confirmed via whole genome sequencing of nasal swabs. For three participants where early nasal swabs were not available, the infecting variant was assigned on the basis of predominant circulating strain at the time of infection. For all participants, whole blood was collected with sodium heparin anticoagulant. Plasma was collected and stored at −80°C, and PBMCs were isolated via Ficoll-Paque separation, cryopreserved in 10% dimethyl sulfoxide/fetal calf serum (FCS), and stored in liquid nitrogen. Study protocols were approved by the University of Melbourne Human Research Ethics Committee (2021-21198-15398-3 and 2056689), and all associated procedures were carried out in accordance with the approved guidelines. All participants provided a written informed consent in accordance with the Declaration of Helsinki.

### Analysis of viral RNA load by qPCR

For viral RNA extraction, 200 μl of nasal swab sample was extracted with the QIAamp 96 Virus QIAcube HT kit (Qiagen, Germany) on the QIAcube HT System (Qiagen) according to the manufacturer’s instructions. Purified nucleic acid was then immediately converted to complementary DNA (cDNA) by reverse transcription with random hexamers using the SensiFAST cDNA Synthesis Kit (Bioline Reagents, UK) according to the manufacturer’s instructions. cDNA was used immediately in the real-time reverse transcription (rRT)–PCR or stored at −20°C. Three microliters of cDNA was added to a commercial real-time PCR master mix (PrecisionFast qPCR Master Mix; Primer Design, UK) in a 20-μl reaction mix containing primers and probes with a final concentration of 0.8 and 0.1 μM for each primer and the probe, respectively. Samples were tested for the presence of SARS-CoV-2 N genes using previously described primers and probes (*28*, *29*). Thermal cycling and rRT-PCR analyses for all assays were performed on the ABI 7500 FAST real-time PCR system (Applied Biosystems, USA) with the following thermal cycling profile: 95°C for 2 min, followed by 45 PCR cycles of 95°C for 5 s and 60°C for 30 s for N gene.

### ELISA (N IgG and blocking ELISA)

Antibody binding to SARS-CoV-2 N protein was tested by ELISA. Ninety-six–well Maxisorp plates (Thermo Fisher Scientific) were coated overnight at 4°C with recombinant N (2 μg/ml). After blocking with 1% FCS in phosphate-buffered saline (PBS), duplicate wells of serially diluted plasma were added and incubated for 2 hours at room temperature. Bound antibody was detected using 1:20,000 dilution of horseradish peroxidase–conjugated anti-human IgG (Sigma-Aldrich), and plates were developed using trimethylboron substrate (Sigma-Aldrich), stopped using sulfuric acid, and read at 450 nm. Endpoint titers were calculated using Graphpad Prism as the reciprocal serum dilution giving signal 2× background using a fitted curve (four-parameter log regression).

For blocking ELISA, plates were coated overnight with recombinant BA.1 or BA.2 spike proteins [Hexapro ectodomain; (*30*)]. Plasma samples were preincubated in 20 μl of PBS with 1 μg of ancestral spike protein or BSA control for 1 hour before serial dilution in PBS and 1% FCS containing ancestral spike protein (5 μg/ml) or BSA. Diluted plasma was added to the coated plate and incubated at room temperature for 30 min, before washing and developing as described above.

### SARS-CoV-2 virus propagation and titration

Ancestral SARS-CoV-2 (VIC01) isolate was grown in Vero cells in serum-free Dulbecco’s modified Eagle’s medium (DMEM) with TPCK trypsin (1 μg/ml), while Omicron BA.1, BA.2, BA.4, XBB, and BQ.1.1 strains were grown in Calu3 cells in DMEM with 2% FCS. Cell culture supernatants containing infectious virus were harvested on day 3 for VIC01 and day 4 for Omicron strains, clarified via centrifugation, filtered through a 0.45-μm cellulose acetate filter, and stored at −80°C. Whole genome sequencing of virus stocks was performed and compared to their respective nasal swab isolate reference genomes. The consensus sequences for each isolate matched the reference genomes apart from BA.1, which had a small in-frame insertion of 9 base pairs in the gene encoding spike (214EPE), and XBB, which had a single sequence change in nsp6 (L3829F).

Infectivity of virus stocks was then determined by titration on HAT-24 cells [a clone of transduced human embryonic kidney–293T cells stably expressing human angiotensin converting enzyme 2 (ACE2) and transmembrane serine protease 2 (TMPRSS2); (*31*)]. In a 96-well flat-bottom plate, virus stocks were serially diluted fivefold (1:5 to 1:78,125) in DMEM with 5% FCS, added with 30,000 freshly trypsinized HAT-24 cells per well, and incubated at 37°C. After 46 hours, 10 μl of alamarBlue Cell Viability Reagent (Thermo Fisher Scientific) was added into each well and incubated at 37°C for 1 hour. The reaction was then stopped with 1% SDS and read on a FLUOstar Omega plate reader (excitation wavelength, 560 nm; emission wavelength, 590 nm). The relative fluorescent units (RFU) measured were used to calculate %viability (“sample” ÷ “no virus control” × 100), which was then plotted as a sigmoidal dose response curve on Graphpad Prism to obtain the virus dilution that induces 50% cell death [50% infectious dose (ID_50_)]. Each virus was titrated in quintuplicate in three to five independent experiments to obtain mean ID_50_ values.

### SARS-CoV-2 microneutralization assay with viability dye readout

In 96-well flat-bottom plates, heat-inactivated plasma samples were diluted 2.5-fold (1:20 to 1:12,207) in duplicate and incubated with SARS-CoV-2 virus at a final concentration of 2× ID_50_ at 37°C for 1 hour. Next, 30,000 freshly trypsinized HAT-24 cells in DMEM with 5% FCS were added and incubated at 37°C. “Cells only” and “virus + cells” controls were included to represent 0 and 100% infectivity, respectively. After 46 hours, 10 μl of alamarBlue Cell Viability Reagent (Thermo Fisher Scientific) was added into each well and incubated at 37°C for 1 hour. The reaction was then stopped with 1% SDS and read on a FLUOstar Omega plate reader (excitation wavelength, 560 nm; emission wavelength, 590 nm). The RFU measured were used to calculate % neutralization with the following formula: (sample − virus + cells) ÷ (cells only − virus + cells) × 100. IC_50_ values were determined using four-parameter nonlinear regression in GraphPad Prism with curve fits constrained to have a minimum of 0% and maximum of 100% neutralization.

### Flow cytometric detection of SARS-CoV-2 spike–reactive B cells

Biotinylated recombinant SARS-CoV-2 spike of ancestral Wuhan Hu-1 and Omicron (BA.1 or BA.2) strains were conjugated to streptavidin-allophycocyanin (APC) or streptavidin–phycoerythrin (PE) fluorophores, respectively. PBMCs were thawed and stained with Aqua viability dye (Thermo Fisher Scientific) and then surface-stained with spike probes: CD19 ECD (J3-119) (Beckman Coulter), IgA VioBlue (IS11-8E10), IgM BUV395 (G20-127), IgD PE-Cy7 (IA6-2), IgG BV786 (G18-145), CD21 BUV737 (B-ly4), CD27 BV605 (O323), streptavidin BV510 (BD Biosciences), CD14 BV510 (M5E2), CD3 BV510 (OKT3), CD8a BV510 (RPA-T8), CD16 BV510 (3G8), and CD10 BV510 (HI10a) (BioLegend). Cells were washed twice with PBS containing 1% FCS and fixed with 1% formaldehyde (Polysciences) and acquired on a BD LSRFortessa using BD FACSDiva.

### Modeling the kinetics of antibody recall and decay

We used a piecewise model to estimate the activation time and growth rate of various immune responses (neutralization and N IgG responses) following breakthrough infections. The model of the immune response *y* for subject *i* at time *y_i_* can be written as

$$y_{i}(t)=\left\{ \begin{array}{c} (B+b_{i});t\geq T_{1}+{\tau_{1}}_{i}\\ (B+b_{i})e^{\left(G+g_{i})[t-(T_{1}+{\tau_{1}}_{i})\right]};T_{1}+{\tau_{1}}_{i}\leq t<T_{2}+{\tau_{2}}_{i}\\ (B+b_{i})e^{\left(G+g_{i})[(T_{2}+{\tau_{2}}_{i})-(T_{1}+{\tau_{1}}_{i})\right]}\times e^{-(D+d_{i})[t-(T_{2}+{\tau_{2}}_{i})]};t\geq T_{2}+{\tau_{2}}_{i} \end{array}\right.$$

The model has five parameters: *B*, *G*, *T*_1_, *D*, and *T*_2_. For a period before *T*_1_, we assumed a constant baseline value *B* for the immune response. After the activation time *T*_1_, the immune response will grow at a rate of *G* until *T*_2_. From *T*_2_, the immune response will decay at a rate of *D*. For each subject *i*, the parameters were taken from a normal distribution, with each parameter having its own mean (fixed effect). A diagonal random-effect structure was used, where we assumed that there was no correlation within the random effects. The model was fitted to the log-transformed data values, with a constant error model distributed around zero with an SD σ. To account for the values less than the LOD, a censored mixed-effect regression was used to fit the model. A categorical covariate was used to quantify the difference in parameters between different groups (i.e., BA.1 versus BA.2 group), and significance was determined on the basis of the value of this binary covariate using a Wald test. Model fitting was performed using MonolixR2019b.

The decay rate of neutralization was estimated by fitting a linear mixed-effect model for each response variable as a function of days after symptom onset, infection wave (i.e., BA.1 and BA.2), and response type (wild type, matched, and BA.4). Likelihood ratio test was used to determine whether the decay rate is different with respect to infection wave and response type. We fitted the model to log-transformed data of various response variables (assuming exponential decay), and we censored the data from below if it was less than the threshold for detection. The model was fitted by using lmec library in *R* (v4.2.1), using the maximum likelihood algorithm to fit for the fixed effects.

### Predicting vaccine efficacy to different variants after breakthrough infection

We used the previously published model for predicting vaccine efficacy (VE) for a given plasma neutralization titer (*11*). This model relates the neutralization titer normalized to the geometric mean neutralization titer against ancestral virus, of a convalescent cohort (individuals infected with the ancestral virus), with VE. Thus, we first estimated the geometric mean peak neutralization titer for BA.1 and BA.2 breakthrough infections against three variants (the ancestral virus, the matched breakthrough variant, and BA.4) and normalized these to a previously reported cohort (*n* = 8) of convalescent individuals (*32*) using the same neutralization assay (geometric mean neutralization titer of this cohort, 507.6). Pooling the data on the decay of neutralization across all individuals and variants, we estimated the kinetics of neutralization against each of these variants up to 3650 days after the peak. These predicted kinetics of neutralization titers were then used in the model by Khoury *et al.* (*11*) to predict VE at each neutralization titer using the formula

$$\mathrm{VE}[n(t)]=\int_{-\infty }^{\infty }N\{x:\log_{10}[n(t)],\sigma \}\;\frac{1}{1+e^{-k\cdot (x-x_{50})}}\;dx$$

where *N*(*x* : μ, α) is the probability density function of a normal distribution with mean μ and SD α, σ is the SD of log_10_ neutralization titers of the vaccinated population, *n*(*t*) is the geometric mean neutralization titer at time *t* days after the peak neutralization titer (normalized to the geometric mean of the convalescent cohort), and *x*_50_ is the log_10_ neutralization titer associated with 50% protection. The parameters σ, *k*, and *x*_50_ were estimated in Khoury *et al.* (*11*) along with SEs of the parameter estimates. Confidence intervals of the predictions were estimated by parametric bootstrapping as described previously (*33*). In brief, 10,000 random samples of the estimated peak neutralization titer; decay rate; and model parameters σ, *k*, and *x*_50_ were randomly drawn from normal distributions around the mean estimates of each parameter using the SE of each estimate or covariance matrix where appropriate, the VE was estimated using the above equation for each set of sampled parameters, and the 2.5th and 97.5th percentiles of the bootstrapped estimates at each neutralization titer were calculated as the lower and upper 95% confidence bounds, respectively (indicated by shaded region in Fig. 4). To account for the values below the LOD, the mean of peak neutralization titer was estimated by fitting a censored normal distribution (using the censReg package in R).
