# Supplementary material for: Durable reprogramming of neutralizing antibody responses following Omicron breakthrough infection
Source: Sci Adv. 2023 Jul 21;9(29):eadg5301. doi: 10.1126/sciadv.adg5301 (PMC10361595; doi:10.1126/sciadv.adg5301)
Supplement: Supplementary file 1 — Figs. S1 to S3 Tables S1 to S4 [file sciadv.adg5301_sm.pdf]

Science Advances
AAAS

Supplementary Materials for

Durable reprogramming of neutralizing antibody responses following Omicron breakthrough infection

Wen Shi Lee *et al.*

Corresponding author: Adam K. Wheatley, a.wheatley@unimelb.edu.au

Sci. Adv. **9**, eadg5301 (2023)
DOI: 10.1126/sciadv.adg5301

This PDF file includes:

Figs. S1 to S3
Tables S1 to S4

Supplementary materials

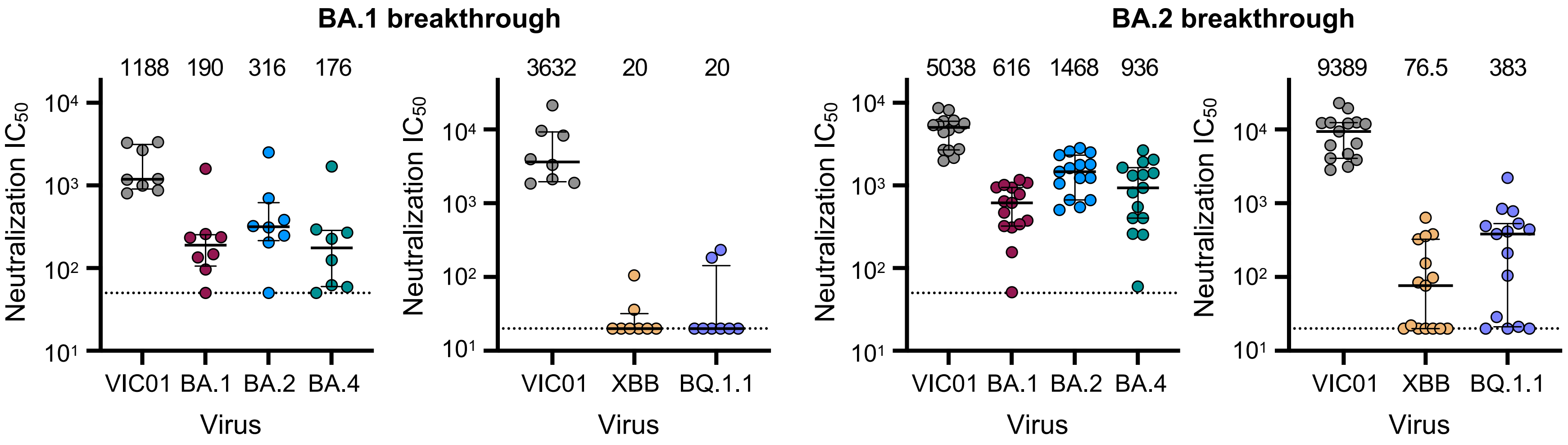

Supplementary Figure S1. Neutralisation mediated by BA.1 and BA.2 breakthrough plasma against ancestral VIC01, Omicron BA.1, BA.2, BA.4, XBB and BQ.1.1 strains at a median of 34 days post-symptom onset. Data are presented as median ± IQR, with median IC_{50} values listed above the data. Neutralisation experiments against (i) VIC01, BA.1, BA.2 and BA.4 and (ii) VIC01, XBB and BQ.1.1 were run on two separate occasions, with data normalised to ancestral VIC01 virus shown in Fig 2B.

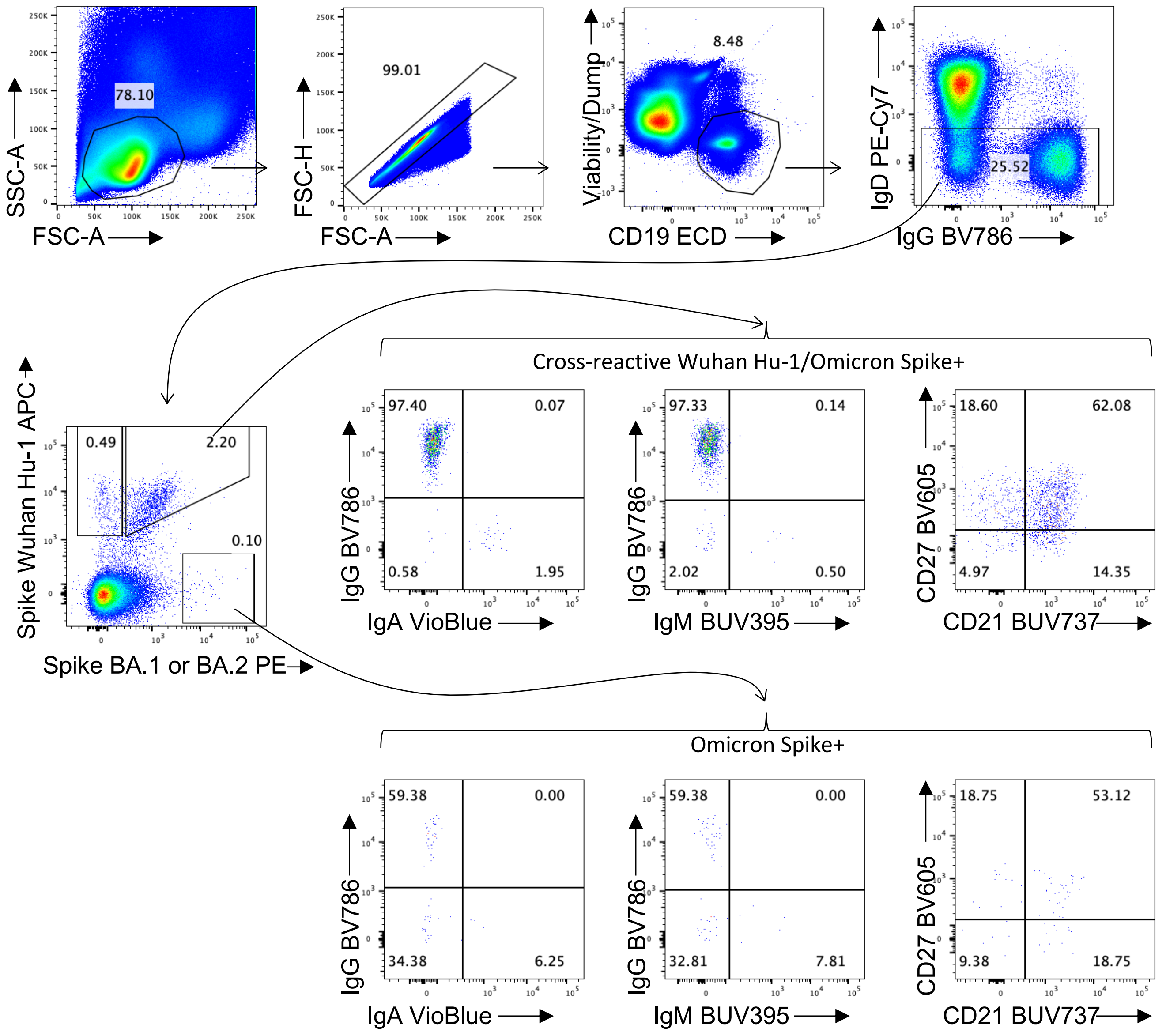

SSC-A
FSC-A
78.10
FSC-H
99.01
Viability/Dump
CD19 ECD
8.48
IgD PE-Cy7
IgG BV786
25.52
Spike Wuhan Hu-1 APC
Spike BA.1 or BA.2 PE
0.49
2.20
0.10
Cross-reactive Wuhan Hu-1/Omicron Spike+
IgG BV786
IgA VioBlue
97.40
0.07
0.58
1.95
IgM BUV395
97.33
0.14
2.02
0.50
CD27 BV605
CD21 BUV737
18.60
62.08
4.97
14.35
Omicron Spike+
59.38
0.00
34.38
6.25
59.38
0.00
32.81
7.81
18.75
53.12
9.38
18.75

Supplementary Figure S2. Gating strategy for the detection and phenotyping of spike-specific B cells. Lymphocytes were identified by FSC-A vs SSC-A gating, followed by doublet exclusion (FSC-A vs FSC-H), and gating on live CD19+ B cells. Class-switched B cells were identified as IgD-. Binding to SARS-CoV-2 ancestral Wuhan Hu-1 or Omicron (BA.1 or BA.2) spike was assessed. Cross-reactive (Wuhan Hu-1+ Omicron+) or mono-specific (Omicron+) B cells were assessed for surface IgM, IgG or IgA isotypes, and CD21 and CD27 co-expression.

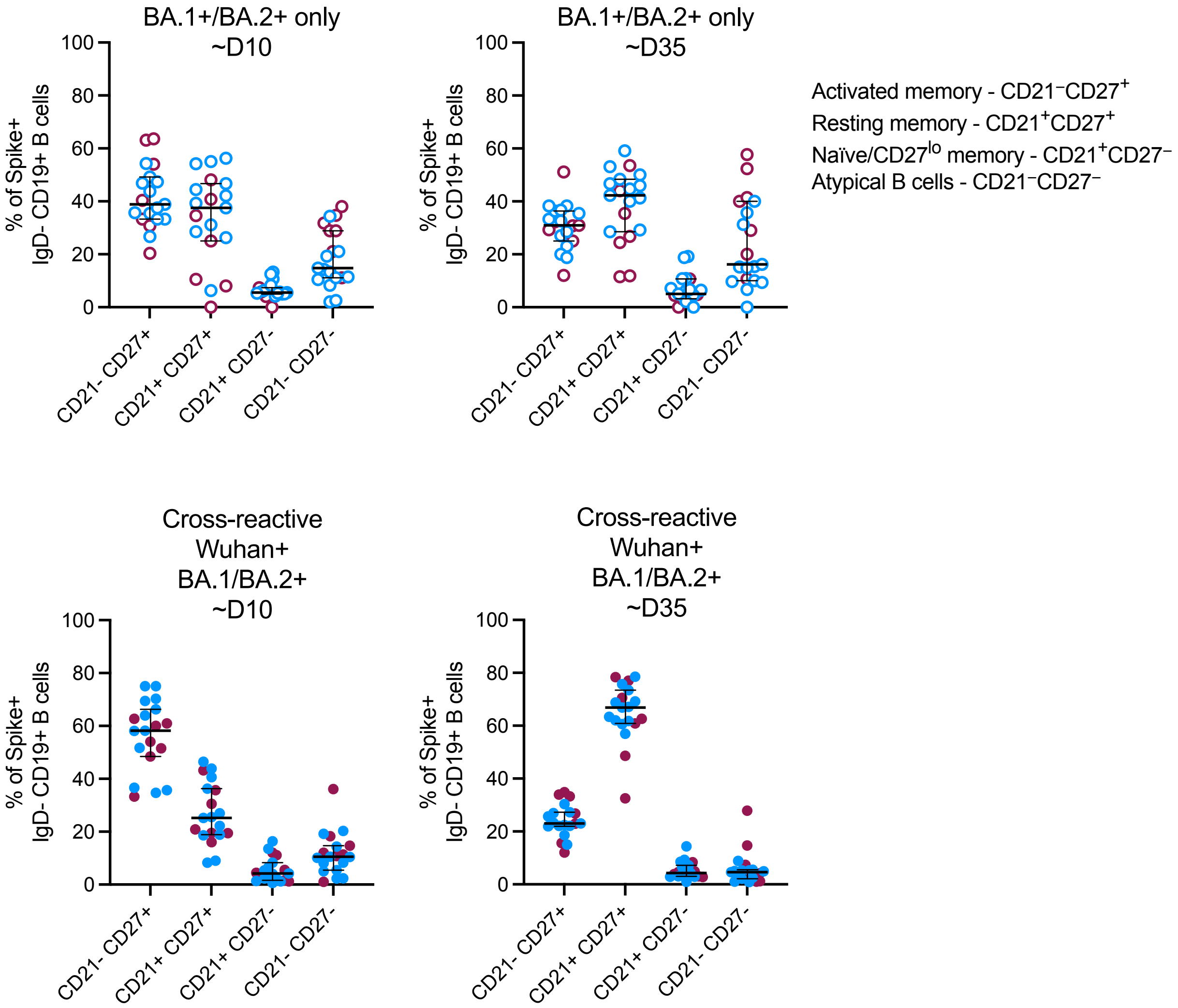
BA.1+/BA.2+ only
~D10
BA.1+/BA.2+ only
~D35
Cross-reactive
Wuhan+
BA.1/BA.2+
~D10
Cross-reactive
Wuhan+
BA.1/BA.2+
~D35
% of Spike+
IgD- CD19+ B cells
0
20
40
60
80
100
CD21- CD27+
CD21+ CD27+
CD21+ CD27-
CD21- CD27-
Activated memory - CD21⁻CD27⁺
Resting memory - CD21⁺CD27⁺
Naïve/CD27lo memory - CD21⁺CD27⁻
Atypical B cells - CD21⁻CD27⁻

Supplementary Figure S3. CD21/CD27 phenotype of Omicron specific (BA.1+ or BA.2+ only) or cross-reactive (Wuhan+ BA.1/BA.2+) B cells at early (~10 days) or late (~35 days) timepoints post- breakthrough infection with Omicron BA.1 (red, n=7) or Omicron BA.2 (blue, n=12). Cross-reactive B cells display early activation (CD21-CD27+) and transition to resting memory phenotype (CD21+CD27+), while Omicron-specific B cells remained constant in their CD21/CD27 profiles.

Supplementary Table 1. Breakthrough infection cohort demographics

Subject	Gender	Age	Breakthrough infection strain	Vaccination/infection history	No. of prior vaccinations and/or infections	Last vaccine to symptom onset (days)	Acute longitudinal sampling (Fig 1B,1C, 2A)	MBC analysis (Fig 3)	Decay analysis (Fig 2C)
COR015	F	27	BA.1	3× BNT162b2	3	38	✓	✓	✓
COR032	F	23	BA.1	2× ChAdOx1 nCoV-19, 1× mRNA-1273	3	32	✓	✓	✓
CP110	M	24	BA.1	2× BNT162b2	2	84	✓	✓	✓
CP111	M	34	BA.1	2× BNT162b2	2	100	✓	✓	
CP112	F	35	BA.1	2× BNT162b2	2	90	✓		
COR198	F	60	BA.1	2× ChAdOx1 nCoV-19, 1× mRNA-1273	3	39	✓	✓	✓
CP069	F	36	BA.1	Infection (Wuhan), 2× BNT162b2	3	111	✓	✓	✓
COR005	M	35	BA.1	1× NVX-CoV2373, 2× BNT162b2	3	102	✓	✓	✓
COR012	F	30	BA.1*	3× BNT162b2	3	38			✓
COR036	F	25	BA.2	2× BNT162b2, 1× mRNA-1273	3	90	✓	✓	
COR274	F	57	BA.2	2× ChAdOx1 nCoV-19, 1× mRNA-1273	3	155	✓	✓	✓
COR275	M	62	BA.2	2× ChAdOx1 nCoV-19, 1× mRNA-1273	3	83	✓	✓	✓
COR291	M	50	BA.2	3× BNT162b2	3	124	✓	✓	
COR039	F	58	BA.2	2× BNT162b2, 1 × mRNA-1273	3	169	✓		✓
COR215	F	55	BA.2	2× ChAdOx1 nCoV-19, 1× mRNA-1273	3	125	✓	✓	✓

COR216	M	58	BA.2	2× ChAdOx1 nCoV-19, 1× mRNA-1273	3	122	✓		
CP120	F	39	BA.2	3× BNT162b2	3	151	✓		
COR043	F	55	BA.2	2× ChAdOx1 nCoV-19, 1× mRNA-1273	3	64	✓	✓	✓
CP116	M	57	BA.2	2× ChAdOx1 nCoV-19, 1× mRNA-1273	3	85	✓	✓	
CP117	M	40	BA.2	3× BNT162b2	3	52	✓	✓	✓
COR281	F	43	BA.2	3× BNT162b2	3	118	✓	✓	
CP118	M	66	BA.2	2× ChAdOx1 nCoV-19, 1× mRNA-1273	3	100	✓	✓	
CP119	F	44	BA.2	2× BNT162b2, 1× mRNA-1273	3	86	✓	✓	
CP40	M	63	BA.2	Infection (Wuhan), 2× ChAdOx1 nCoV-19, 1× BNT162b2	4	49	✓	✓	
COR011	F	35	BA.2*	3× BNT162b2	3	125			✓
COR282	M	30	BA.2*	3× BNT162b2	3	157			✓

*Nasal swabs to determine infecting variant were unavailable, thus infecting strain was assigned by predominant circulating strain at the time of infection.

Supplementary Table 2. Piecewise linear regression parameters of N-binding IgG (with 95% CI) following BA.1 or BA.2 breakthrough infection. Related to Fig 1B.

N IgG	Estimated parameter (95% CI)		
	BA.1 vs BA.2		
	BA.1	BA.2	p-value (for the difference)
Activation time (days post onset)	7.4 (5.4 - 8.7)	6.7 (3.4 - 9.6)	0.63
Doubling time (days)	2.4 (1.9 - 3.7)	1.5 (0.9 - 4)	0.08
Fold change (from baseline)	9 (1.6 - 16.5)	14.5 (7.9 - 21)	0.24

Supplementary Table 3. Piecewise linear regression parameters of neutralising antibodies (with 95% CI) following BA.1 or BA.2 breakthrough infection. Related to Fig 2A.

Neutralization IC_{50}	Estimated parameter (95% CI)								
	BA.1 breakthrough infection			BA.2 breakthrough infection			BA.1 vs BA.2		
	WT	BA.1	p-value (for the difference)	WT	BA.2	p-value (for the difference)	BA.1	BA.2	p-value (for the difference)
Activation time (days post onset)	2.3 (0.3 - 20.3)	3.1 (1.5 - 6.6)	0.82	2.3 (0.8 - 7.2)	3.6 (2.4 - 5.6)	0.55	3.1 (1.5 - 6.6)	3.6 (2.4 - 5.6)	0.78
Doubling time (days)	6.1 (3.3 - 11.3)	2.1 (1.5 - 3)	0.02	7.8 (5.4 - 11.2)	2.8 (2.3 - 3.5)	0.000013	2.1 (1.5 - 3)	2.8 (2.3 - 3.5)	0.26
Fold change (from baseline)	5.4 (2.2 - 8.7)	31 (13.7 - 48.3)	0.0078	15.6 (6.5 - 37.8)	34.7 (11.9 - 57.4)	0.0042	31 (13.7 - 48.3)	34.7 (11.9 - 57.4)	0.53

Supplementary Table 4. Decay rates of neutralising antibodies (with 95% CI) following BA.1 or BA.2 breakthrough infection. Related to Fig 2C.

Neutralization IC_{50}	Estimated parameter (95% CI)								
	BA.1 breakthrough infection			BA.2 breakthrough infection			BA.1 vs BA.2		
	WT	BA.1	p-value (for the difference)	WT	BA.2	p-value (for the difference)	BA.1	BA.2	p-value (for the difference)
Half-life (days)*	182.8 (110.7 - 523.8)	333.5 (136.9 - 10000)	0.98	1216 (252.6 - 10000)	1049.7 (210.5 - 10000)	0.43	333.5 (136.9 - 10000)	1049.7 (210.5 - 10000)	0.36

*half-life upper limit is set to 10000 days
